# Airway management in “tubeless” spontaneous-ventilation video-assisted thoracoscopic tracheal surgery: a retrospective observational case series study

**DOI:** 10.1186/s13019-023-02157-w

**Published:** 2023-02-04

**Authors:** Yuying Liu, Lixia Liang, Hanyu Yang

**Affiliations:** Department of Anesthesia, The First Affiliated Hospital of Guangzhou Medical University, No. 151 Yanjiang Rd, Guangzhou, 510120 China

**Keywords:** Airway management, Spontaneous-ventilation video-assisted thoracoscopic surgery, Tracheal surgery

## Abstract

**Background:**

Surgeon and anesthetist share the airway in a simpler way in the resection and reconstruction phase of tracheal surgery in tubeless spontaneous-ventilation video-assisted thoracoscopic surgery (SV-VATS). Tubeless SV-VATS means stable spontaneous ventilation in the resection and reconstruction phase to anesthesiologist, and unobstructed surgical field to surgeon. What’s the ideal airway management strategy during “Visual Field tubeless” SV-VATS for tracheal surgery is still an open question in the field.

**Methods:**

We retrospectively reviewed 33 patients without sleeve and carina resections during the study period (2018–2020) in our hospital. The initial management strategy for these patients was spontaneous ventilation for intrathoracic tracheal resection and reconstruction. We obtained and reviewed medical records from our institution’s clinical medical records system to evaluate the airway management strategy and device failure rate for tracheal resection in Tubeless SV-VATS.

**Results:**

Between 2018 and 2020, SV-VATS was first attempted in the 33 patients who had intrathoracic tracheal surgery but without sleeve and carina resections. All patients underwent bronchoscopy (33/33) and 8 patients (8/33) received partial resection before surgery. During the surgery, the airway device comprised either a ProSeal laryngeal mask airway (ProSeal LMA) (n = 27) or single lumen endotracheal tube (n = 6). During the resection and reconstruction phase, Visual Field tubeless SV-VATS failed in 9 patients, and breathing support switched to plan B which is traditional ventilation of a single lumen endotracheal tube for cross field intubation (n = 4) and ProSeal LMA alongside a high-frequency catheter (high-frequency jet ventilation, HFJV) (n = 5) into the distal trachea ventilation. Preoperative respiratory failure or other ventilation-related complications were not observed in this cohort.

**Conclusion:**

Base on current analysis either ProSeal LMA or endotracheal tube is an effective airway management strategy for tubeless SV-VATS with appropriate patient selection. It also provides breathing support conversion option when there’s inadequate ventilation.

## Background

Changes in surgical practice have resulted innovations in video-assisted thoracic surgery (VATS) and enhanced recovery after surgery (ERAS). Studies by other surgeons and attempts at Visual Field tubeless have provided a good basis for the development of VATS [1–4].

SV-VATS has recently been reported to be an effective and feasible technique in several centers and articles [1, 2, 5]. It was first performed with simple pleural and lung procedures, and then progressed to pulmonary resections, sleeve resection and tracheal and carinal resections. Consistent intravenous sedation and analgesia are necessary, along with epidural or intercostal nerve block, incision regional anesthesia[2, 6] or vagal nerve block to offer complete pain control to maintain stable spontaneous ventilation and relieve the cough reflex [3, 7, 8].

The use of tubeless SV-VATS in the resection phase under stable spontaneous ventilation could reduce the apneic time required to perform airway reconstruction, and provide an unobstructed surgical field because the surgeon and anesthetist share the airway in a simpler way. There have been several published case series which reported the advantages of using SV-VATS as a novel approach in TRR [9, 10]. Airway management of tracheal surgery divides into 3 stages and different airway management strategies were introduced [11, 12]. Van Regemorte et al. [13] reported that controlled ventilation through the Rusch flexible intubation guide catheter showed satisfactory and stable ventilatory parameters in two patients. The tracheal tubes obstruct access to the posterior tracheal wall and are prone to causing accidental surgical damage to the cuff. Extracorporeal membrane oxygenation (ECMO) was considered as a more invasive alternative or only as a rescue device [14–16]. These problems can be simplified by SV-VATS. However, because it’s challenging to perform SV-VATS tracheal surgery, there’s risk of ventilation failure during the resection and reconstruction phase of tubeless tracheal resection. Although the use of SV-VATS is increasing, there’s still no instructive protocol available for airway management in these patients.

To better understand tubeless SV-VATS for TRR, this report reviewed airway management experience for intrathoracic tubeless tracheal resection from 2018 to 2020 in our center.

## Methods

This study was approved by the First Hospital of Guangzhou Medical University Research Ethics Committee, and written informed consent was waivered. We retrospectively collected and reviewed medical records of all patients treated at our hospital from 2018 to 2020, who were diagnosed with tracheal stenosis, who underwent TRR and for whom the initial strategy was spontaneous ventilation, without main bronchial sleeve and carina resections.

**Fig. 1:**
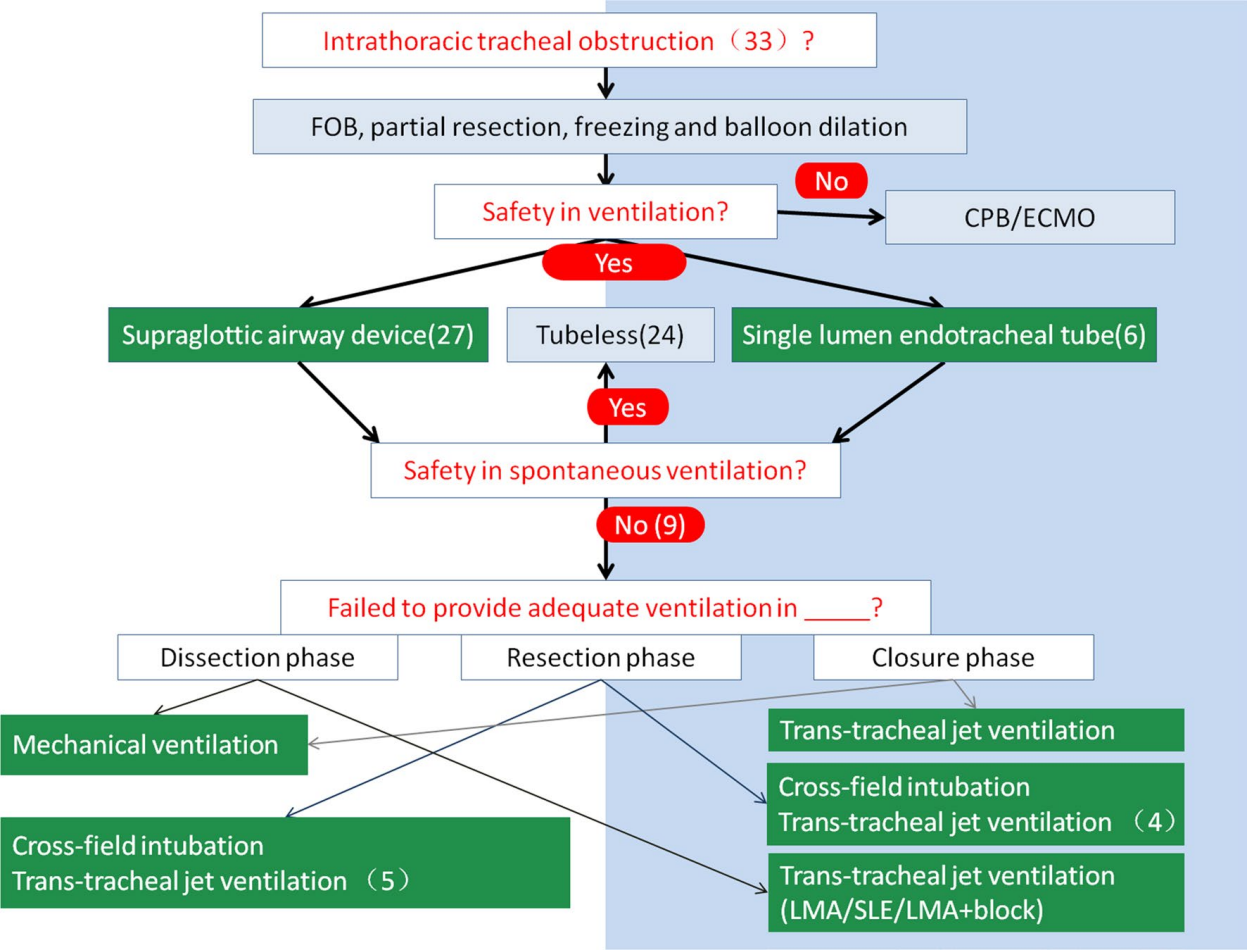
Spontaneous ventilation was first attempted in 33 patients with intrathoracic tracheal stenosis. The airway device was chosed by the attending anesthetist and comprised either a laryngeal mask airway (ProSeal LMA) (n = 27) or single lumen endotracheal tube (n = 6). During the resection and reconstruction phase, “tubeless” failed in 9 patients and they used plan B: which is switch to traditional ventilation

When a patient came to our hospital and was suspected of having tracheal stenosis, a high-resolution computerized tomography (CT) scan of the neck and upper thorax were performed. Tracheal lesions were evaluated by computed tomography images. Examination with a fiber optic bronchoscope (FOB), biopsies and histological diagnosis were carried out in all patients except those with iatrogenic subglottic tracheal stenosis. If there was more than 70–80% obstruction of the trachea along with aggravation of exertion dyspnea, intervention dilation or laser coagulation through FOB was used preoperatively (Fig. 1).

After stricter patient selection, intraoperative management of the patients was provided by an experienced anesthesiologist. Multidisciplinary consultation was held to assess risks and benefits. Careful choreographing of each step and efficient teamwork between anesthesiologists, operating room (OR) staff and surgeons were carried out to ensure the patient safety.

For selected patients the standard monitors were placed, bispectral index (BIS) was used to monitor anesthetic depth to maintain adequate sedation levels, a central venous line (in cases in which the need for potent vasoactive drugs is anticipated, the subclavian or internal jugular approach was optional and should be away from the surgical field) and an intra-arterial catheter (preferably in the radial artery, for hemodynamic changes and arterial blood gas monitoring) were also needed for this operation.

Epidural anesthesia, thoracic paravertebral block or intercostal nerve blocks were performed. After that, anesthesia was induced with target-controlled infusion (TCI) of propofol. A laryngeal mask airway (LMA) or well-lubricated endotracheal tube was placed according to standard technique. The choice of airway device was reviewed.

All those patients we focused on were treated by tracheal resection with end-to-end anastomoses, which was mainly divided into three distinct phases: the dissection phase, the resection phase (incision/ resection and reanastomosis of the airway) and the closure phase. Normally, spontaneous respiration was maintained before completion of the anastomosis. To surgeons, “tubeless” means tubeless in the resection phase under stable spontaneous ventilation. During the resection and reconstruction phases, supplemental oxygen was provided via the airway device to maintain an adequate oxygenation supply. If spontaneous respiration failed to provide adequate ventilation, airway management would be changed to conventional airway management approaches and traditional ventilation models, and crossfield intubation or high frequency ventilation (HFV) would be used. We reviewed the device failure rate to evaluate the safety feature for ProSeal LMA and endotracheal tube respectively.

Patient demographics, etiology, location and morphology of the stenotic area, preoperative treatment, airway device and device failure, functional status, anesthetic management, medical therapy, hemodynamic measurements, clinical course and outcomes, blood chemistry, and capillary blood gas analysis were reviewed.

Normally-distributed data were expressed as mean ± standard deviation. Non-normally distributed data were expressed as median and interquartile range. Data analysis was performed using SPSS version 22.0 (SPSS, Inc, IBM Inc., Armonk, NY, USA).

Anesthetic agents were almost the same for intravenous analgesia and sedation. Anesthesia was maintained with propofol (target plasma concentration of 1.5–3.0 μg/mL), dexmedetomidine 0.5–1 μg/kg/h, and remifentanil 0.01–0.05 μg/kg/min. In these procedures, anesthetists try to reduce neuromuscular blockade, but not to stop it completely. A small dose of muscle relaxant was chosen in several patients with diaphragmatic contraction and pendelluft. We tested the ability to ventilate spontaneously after each administration until the ventilation and surgical field was balanced. The patient was ventilated with 100% oxygen via the airway device under spontaneous breathing; the dosage of muscle relaxant in the resection phase is presented in Table 2. Furthermore, dopamine, or norepinephrine was used to maintain cardiac output and systemic blood pressure perioperatively. Unfortunately, we did not record the rate of cough reflex and the vigorous mediastinal movement which will be recorded in our future study.

Thoracic epidural anesthesia (TEA) or other local anesthesia was administered in the operation room before anesthesia induction. Vagal block was performed adjacent to the vagus nerve. Topical lidocaine was applied to the surface of the lung before surgery under direct thoracoscopic vision. The choice of local anesthetic is presented in Table 2.

## Results

1. Baseline characteristics

Between 2018 and 2020, spontaneous ventilation was first attempted in 33 patients who had been diagnosed with primary tracheal tumor/neoplasm preoperatively; sleeve and carina resections were not included.

Baseline demographic characteristics are depicted in Table 1. The average age was 44.3 ± 12.9 years. The average size of the tracheal lesion was 11 ± 6.9 mm and the narrowest tracheal diameter was approximately 0.4 mm with a crescent-shaped channel to breathe. Patients had an average extent of approximately 7.9 ± 14.3 cm distal to the vocal cords. All patients underwent bronchoscopy (33/33) and balloon dilation or partial resection (8/33) before surgery during preoperative preparation.

2. Airway management and ventilation models

**Table 1:** Baseline characteristics of patients with tracheal stenosis who received tracheal resection

Patient	Sex	Age (44.3 ± 12.9)	Height (164.1 ± 7.9)	Body weight (62.7 ± 13)	BMI (23.2 ± 3.7)	Comorbidity	Smoking history	Preoperative treatment	Beginning distance below the glottis (7.9 ± 14.3 mm)	Sectional area of the tumor within trachea(%)	Transverse diameter of airway stenosis (11 ± 6.9 mm)
1	Male	25	170	59	20.4	No	Yes, 2 years	Resection with bronchoscopy	60	26.83	9
2	Female	58	154	48	20.2	Diabete	No	Bronchoscopy	80	72.73	6
3	Male	37	169	70	24.5	No	No	Resection with bronchoscopy	70	36.05	11
4	Male	20	163	56	21.1	No	No	Bronchoscopy	44	32.19	9.9
5	Female	74	156	60	24.7	COPD	No	Bronchoscopy	75	46.05	8.2
6	Female	48	160	48	18.7	Gastritis	No	Bronchoscopy	72	85.33	6
7	Female	31	170	55	19.0	No	No	Bronchoscopy	75	86.49	4
8	Female	40	160	57	22.3	Epilepsy	No	Bronchoscopy	86	77.30	3.7
9	Male	72	167	52	18.6	No	No	Bronchoscopy	88	69.14	7.5
10	Female	44	152	50	21.6	No	No	Resection with bronchoscopy	78	10.83	10.7
11	Female	37	160	50	19.5	No	No	Bronchoscopy	60	77.94	3
12	Male	62	155	50	20.8	COPD	No	Bronchoscopy	50	44.72	11
13	Male	48	166	82	29.8	No	No	Resection with bronchoscopy	90	41.50	2
14	Female	31	156	68	27.9	No	No	Resection with bronchoscopy	69	70.20	4.5
15	Male	50	165	75	27.5	No	NO	Bronchoscopy	90	17.40	19
16	Male	36	169	70	24.5	NO	NO	Resection with bronchoscopy	70	36.00	3.6
17	Male	54	170	43	14.9	NO	YES	Bronchoscopy	94	48.24	20
18	Male	52	165	64	23.5	NO	NO	Bronchoscopy	88	25.92	24
19	Male	36	169	70	24.5	NO	NO	Bronchoscopy	108	25.71	18
22	Male	41	160	64	25	NO	NO	Resection with bronchoscopy	78	66.67	17
23	Male	50	165	75	27.6	NO	NO	Bronchoscopy	100	22.72	20
24	Female	53	151	58	25.4	NO	NO	Resection with bronchoscopy	82	70	8
26	Female	40	165	63	23.1	NO	NO	Bronchoscopy	82	24.86	13
27	Male	57	162	59	22.5	Cerebral infarction	NO	Bronchoscopy	76	18.8	20
28	Female	31	160	46	17.9	NO	NO	Bronchoscopy	91	61.11	2
29	Male	40	175	85	27.8	Asthma,smoking 10 years	YES, 10	Bronchoscopy	86	27.47	10
30	Male	50	170	85	29.4	Hypertension	NO	Bronchoscopy	89	72.73	15
32	Male	31	165	62	22.8	Hepatitis B	NO	Bronchoscopy	91	25	7
33	Male	38	190	94	26	NO	NO	Bronchoscopy	94	57.14	26

In the operating theater, the patient was comfortably positioned on the operating table and don’t having difficulty in mask ventilation after induction of anesthesia. 33 patients successfully completed the surgery in the operating room. All the patients were tried with spontaneous respiration initially under sedation throughout the surgery. Eventually, in 24 patients (24/33) the surgery was completed by tubeless SV-VAT in the resection phase under stable spontaneous ventilation as depicted in Table 2. The average operation time was 255.1 ± 80.7 min.

**Table 2:** Operative information of patients with intrathoracic tracheal stenosis who received tracheal resection

Patient	Airway management	Change of airway management	Resection-Phase	Local anesthesia	Neuromuscular blocker use	Surgery time (255.1 ± 80.7 min)	Anesthesia time (357.1 ± 83.9 min)	Preoperative PO_2_ (108.3 ± 33.2)	Preoperative PCO_2_ (41.8 ± 4.5)	Preoperative Lac (1.5 ± 0.6)	APACHE 2 score (4.4 ± 3.2)	ICU stay (1.3 ± 1.0)	Postoperative complications
1	LMA	No	SRA	1	≥ 1 ED95	180	240	90	42	1.8	5	1	
2	LMA	No	SRA	1	0	192	318	79	51.7	1.07	4	1	
3	LMA	CRO	SIMV	1	0	300	420	90.2	42.5	1.77	4	1	
4	LMA	No	SRA	2	≥ 1 ED95	240	396	139.6	38.1	1.2	4	1	
5	SLE	CRO	SIMV	1	0	120	240	78.6	38.7	2.66	14	1	
6	LMA	No	SRA	1	0	264	342	163	38	1.69	5	1	
7	LMA	No	SRA	2	≥ 1 ED95	258	312	121	43	1.35	0	1	
8	LMA	No	SRA	1	≥ 1 ED95	300	540	159	27	1.5	5	1	
9	LMA	HFJV	HFJV	1	≥ 1 ED95	270	276	67.9	33.6	1.5	12	1	
10	LMA	No	SRA	1	0	162	276	88.4	41.6	1.6	6	1	
11	SLE	CRO	SIMV	1	≥ 1 ED95	516	540	88.4	42.3	1.87	2	6	Anastomosis dehiscence
12	SLE	No	SRA	1	≥ 1 ED95	210	360	81.4	45.3	1.54	6	2	Atrial fibrillation
13	LMA	HFJV AND CRO	HFJV	1	≥ 1 ED95	348	450	66.1	42.4	2.86	4	1	
14	SLE	HFJV	HFJV	1	≥ 1 ED95	168	258	126.2	42.6	1.01	6	1	
15	LMA	No	SRA	1	0	198	300	144.5	41.7	1.1	5	1	
16	LMA	No	SRA	1	0	318	408	167	41.8	0.93	9	1	
17	LMA	No	SRA	1	0	222	318	79.1	44.8	1.05	4	2	
18	LMA	No	SRA	1	≥ 1 ED95	234	318	145.8	40.2	1.17	2	1	
19	LMA	No	SRA	1	0	228	312	167	41.8	0.93	0	1	
22	LMA	No	SRA	1	0	192	318	97.9	41.3	1.01	2	1	
23	LMA	No	SRA	1	0	192	300	145	41.7	1.11	5	1	
24	SLE	HFJV	HFJV	1	0	270	384	68.4	44	1.86	4	1	
26	LMA	No	SRA	1	≥ 1 ED95	276	390	106	44.9	0.94	3	1	
27	LMA	CRO	SIMV	1	≥ 1 ED95	330	438	79.4	44.2	1.73	5	1	
28	LMA	No	SRA	1	0	162	246	138.9	47.8	0.83	1	1	
29	LMA	No	SRA	1	0	360	480	96.2	43.1	2.51	5	2	
30	SLE	No	SRA	1	0	354	444	85.3	36.7	1.36	4	2	
32	LMA	No	SRA	1	0	240	330	90.1	42.8	1.41	0	0	
33	LMA	HFJV	HFJV	1	0	294	402	92.7	47.5	3.15	1	2	

*SLE* Single lumen endotracheal change of airway management, *CRO* crossfieid intubation. Local anesthesia: 1, Epidural anesthesia; 2, paravertebral block

ProSeal LMA (27 patients) or a single lumen endotracheal tube (6 patients) was selected in 33 patients. 24 patients were managed successfully under spontaneous respiration during resection and re-anastomosis of the trachea. Instability of spontaneous ventilation resulted in oxygen desaturation in 9 cases (5 patients used ProSeal LMA and 4 patients performed a single lumen endotracheal tube) during dissection and resection of the airway, failure of tubeless SV-VAT and a switch to mechanical ventilation throughout the operation. Elective cross field intubation was then used by the surgical staff during resection and anastomosis of the airway. Before the trachea was opened, we inserted a jet catheter (A type guide wire hollow type, WELL LEAD MEDICAL CO., LTD, 20182021075) through an LMA or single lumen endotracheal tube. The catheter passed through the stenosis with high frequency ventilation used. After the trachea was opened, cross field intubation was used. After complete airway separation, cross field intubation was also helpful. Those processes are depicted in Fig. 1.

Mechanical ventilation support was available for all patients in the closure phase, including SIMV mode that is compatible with spontaneous breathing. All patients resumed spontaneous breathing in the anesthesia resuscitation room, without ventilation support. They were sent to ICU for future observation to minimize postoperative risk.

3. Postoperative complications and outcomes

Postoperatively, surgical complications occurred in two patients: anastomosis dehiscence (No. 11), and atrial fibrillation within 24 h after surgery (No. 12). Preoperative respiratory function index and APACHE 2 scores regarding patient quality of life are presented in Table 2. The average APACHE 2 score was 4.4 ± 3.2 and the average ICU stay was 1.3 ± 1.0 days. There was no case of preoperative respiratory failure or other ventilation-related complications. No intraoperative complications, including persistent hypercarbia, laryngeal edema or hoarseness related to anesthetic management were reported.

## Discussion

Airway management in tracheal stenosis is a controversial topic in clinical practice [17, 18]. The goals for airway management are to maintain a secure and unobstructed airway (tubeless) and to provide optimal access and surgical conditions. Tubeless SV-VATS holds the promise of a valuable approach for thoracoscopic surgery [19, 20]. Surgeon and anesthetist share the airway in a simpler way so an obvious benefit of SV-VATS in the resection phase of tracheal surgery is improved visual field and unobstructed surgical field. The procedure with Visual Field tubeless provides reduced surgical field interference and the operator can focus on the surgery. This is an innovative attempt in tracheal surgery.

In the application of any new procedure, the safety of the patients is always of paramount importance. Several studies reported SV-VATS in patients who were relatively young and not obese, with good health and no complex anatomy [21, 22]. This may introduce patient selection bias. However these reports indicate that SV-VATS is at least as safe and efficient as traditional anesthesia with appropriate patient selection [23], although evidence from available studies about SV-VATS remains sparse. Thus, when starting SV-VATS tracheal reconstruction, proper patient selection, adequate airway management experience, and preferably a certain amount of training are recommended to minimize complications and conversions. Disease features and patients’ characteristics, as well as efficient teamwork between anesthesiologists, operating room (OR) staff and surgeons are absolutely necessary. Nevertheless, to enhance our sensitivity to the potential complications, all patients were sent to the ICU after meeting the PACU discharge criteria and without breath support. No case of postoperative respiratory failure or other ventilation-related complication was reported.

In this trial, 33 patients were analyzed. We showed that a supraglottic airway device or endotracheal tube were both alternative airway options as traditional airway devices. Tubeless is better but the conventional airway techniques for tracheal stenosis is the technology-bases [12, 24]. When there are unstable spontaneous-ventilation condition and difficulty in progression in the resection and reconstruction phase, conversion to traditional mechanical ventilation is necessary to ensure patient safety. In such case, plan B: ProSeal LMA alongside a high frequency catheter or cross field intubation was selected [25, 26]. The device failure rate was 27.3% (9/33). The develop of new devices for airway management (eg, igel, more and better intravenous anesthetics such as dexmedetomidine) [27, 28], and stricter patient selection will help decrease the device failure rate of this surgery when ERAS of VATS was just beginning [29, 30]. Nevertheless, in this review, instability of spontaneous breath resulted in failure of tubeless during dissection and resection of the airway in 9 cases were smoothly processed for respiratory support conversion. Limited by the number of cases, serious complications such as pulmonary aspiration, early postoperative bleeding or suture dehiscence were not observed in this cohort.

LMA was considered as a feasible alternative during open tracheal surgery, and was still used in several studies of SV-VATS [25, 31]. As a superior glottis airway can’t be affected by narrow airways, it has a unique advantage in cases when the tumors are near the glottis or stenosis after intubation. This device is effective for various types of airway surgery, presents excellent clinical outcomes and has been recommended for use in cervical trachea reconstruction or cases near the glottis. In some patients, a single-lumen endotracheal catheter can be selected if the stenosis area is far away from the glottis [32, 33]. We selected this option for obese patients but high quality randomized trials are recommended to further objectify this decision. Kashii et al. reported that after ETT insertion, a patient maintained spontaneous respiration without any hypoxic event [27]. For patients with high BMI, high risk of regurgitate or other factors that may cause unstable spontaneous breath, some anesthetists chose a single-lumen endotracheal tube because an LMA cannot ventilate with high pressure. However, use of endotracheal tube may be impossible if the stenotic segment is too high and too close to the glottis. On the other hand, given that there’s difference in airway management preferences by the anesthesiologist, whether these potential benefits can be proved in the future by well-designed RCTs are not known.

Even if current SV-VATS offers some potential advantages, technical difficulties including diaphragmatic contraction and pendelluft, airway hyperreactivity and cough, reverse trigger and breath stack during mechanical ventilation still need to be overcome [34]. Moreover, in an attempt to minimize non-uniform transmission of pleural pressure generated by diaphragmatic contraction under spontaneously breath in the resection phase, several patients used a small dose of muscle relaxant to balance diaphragmatic contraction of spontaneous ventilation and mediastinal swing in surgical field.

In conclusion, our experiences suggested that either supraglottic airway device or endotracheal tube is a feasible and prospective strategy for tubeless TRR. They also provide options for the conversion to traditional ways under the condition of unstable spontaneous ventilate. A sample size of 33 patients will not allow a comprehensive safety evaluation, especially with regards to rare TRR surgery. Given the small number of cases examined, limitations of applied indications, contraindications, and per-operative period safety warrant further investigation. However, large patient cohorts are difficult to evaluate, as tracheal resections are relatively rare, even in specialized centers. This method needs more evidence before being recommended for potential guidelines.

## Conclusion

Either supraglottic airway device or endotracheal tube is an effective strategy for tubeless SV-VATS with appropriate patient selection. It also, provides breathing support conversion option when there’s inadequate ventilation.

## Data Availability

All data generated or analyzed during this study are included in this published article.

## Abbreviations

VATS: Video-assisted thoracic surgery
ERAS: Enhanced recovery after surgery
SV-VATS: Spontaneous-ventilation video-assisted thoracoscopic surgery
TRR: Tracheal resection and resections
FOB: Fiber optic bronchoscope
ProSeal LMA: ProSeal laryngeal mask airway
HFV: High frequency ventilation
TEA: Thoracic epidural anesthesia

## References

[1] Hung WT, Hung MH, Wang ML, Cheng YJ, Hsu HH, Chen JS (2019). Nonintubated thoracoscopic surgery for lung tumor: seven years' experience with 1,025 patients. Ann Thorac Surg.

[2] Akopov A, Kovalev M (2020). Nonintubated tracheal surgery. Thorac Cardiovasc Surg.

[3] Hung WT, Cheng YJ, Chen JS (2020). Video-assisted thoracoscopic surgery lobectomy for lung cancer in nonintubated anesthesia. Thorac Cardiovasc Surg.

[4] Sastre I, Espana M, Ceballos RJ, Bustos MEF: VATS tracheal resection and reconstruction. *Multimedia manual of cardiothoracic surgery : MMCTS* 2020, 2020. 10.1510/mmcts.2020.069 33471451

[5] Kao MC, Lan CH, Huang CJ (2012). Anesthesia for awake video-assisted thoracic surgery. Acta Anaesthesiol Taiwanica : Off J Taiwan Soc Anesthesiol.

[6] Hung M-H, Hsu H-H, Chan K-C, Chen K-C, Yie J-C, Cheng Y-J, Chen J-S (2014). Non-intubated thoracoscopic surgery using internal intercostal nerve block, vagal block and targeted sedation. Eur J Cardiothorac Surg.

[7] Li S, Liu J, He J, Dong Q, Liang L, Yin W, Pan H, He J (2016). Video-assisted thoracoscopic surgery resection and reconstruction of thoracic trachea in the management of a tracheal neoplasm. J Thorac Dis.

[8] Guo Z, Yin W, Wang W, Zhang J, Zhang X, Peng G, Xu X, Huang Z, Liang L, Chen H (2016). Spontaneous ventilation anaesthesia: total intravenous anaesthesia with local anaesthesia or thoracic epidural anaesthesia for thoracoscopic bullectomy. Eur J Cardio-Thorac Surg Off J Eur Assoc Cardio-Thorac Surg.

[9] Caronia FP, Loizzi D, Nicolosi T, Castorina S, Fiorelli A (2017). Tubeless tracheal resection and reconstruction for management of benign stenosis. Head Neck.

[10] Schieren M, Bohmer A, Dusse F, Koryllos A, Wappler F, Defosse J (2017). New approaches to airway management in tracheal resections-a systematic review and meta-analysis. J Cardiothorac Vasc Anesth.

[11] Auchincloss HG, Mathisen DJ (2018). Tracheal stenosis-resection and reconstruction. Ann Cardiothor Surg.

[12] Smeltz AM, Bhatia M, Arora H, Long J, Kumar PA (2020). Anesthesia for resection and reconstruction of the trachea and carina. J Cardiothorac Vasc Anesth.

[13] Van Regemorter V, Potie A, Schmitz S, Scholtes JL, Veevaete L, Van Boven M (2018). Successful ventilation through a Rusch intubation guide catheter in severe laryngotracheal stenosis. J Otolaryngol Head Neck Surg Le Journal d'oto-rhino-laryngologie et de chirurgie cervico-faciale.

[14] Sanchez-Lorente D, Iglesias M, Rodriguez A, Jungebluth P, Macchiarini P (2012). The pumpless extracorporeal lung membrane provides complete respiratory support during complex airway reconstructions without inducing cellular trauma or a coagulatory and inflammatory response. J Thorac Cardiovasc Surg.

[15] Stoica RT, Cordos I, Popescu WM (2020). Anesthetic considerations for tracheobronchial resection in oncologic surgery. Curr Opin Anaesthesiol.

[16] Lang G, Ghanim B, Hotzenecker K, Klikovits T, Matilla JR, Aigner C, Taghavi S, Klepetko W (2015). Extracorporeal membrane oxygenation support for complex tracheo-bronchial proceduresdagger. Eur J Cardio-Thorac Surg Off J Eur Assoc Cardio-Thorac Surg.

[17] Wiedemann K, Mannle C (2014). Anesthesia and gas exchange in tracheal surgery. Thorac Cardiovasc Surg.

[18] Ahuja S, Cohen B, Hinkelbein J, Diemunsch P, Ruetzler K (2016). Practical anesthetic considerations in patients undergoing tracheobronchial surgeries: a clinical review of current literature. J Thorac Dis.

[19] Liu J, Li S, Shen J, Dong Q, Liang L, Pan H, He J (2016). Non-intubated resection and reconstruction of trachea for the treatment of a mass in the upper trachea. J Thorac Dis.

[20] Okuda K, Nakanishi R (2016). The non-intubated anesthesia for airway surgery. J Thorac Dis.

[21] Wang C, Wu D, Pang P, Kong H, Zhao J, Chen X, Ye J, Pan Z, Liang W, Liu J *et al*: Spontaneous Ventilation Video-Assisted Thoracoscopic Surgery for Geriatric Patients With Non-Small-Cell Lung Cancer. *J Cardiothorac Vasc Anesthesia* 2021. 10.1053/j.jvca.2021.07.042 34419362

[22] Guido-Guerrero W, Bolaños-Cubillo A, González-Rivas D (2018). Single-port video-assisted thoracic surgery (VATS)-advanced procedures & update. J Thorac Dis.

[23] Sunaga H, Blasberg JD, Heerdt PM (2017). Anesthesia for nonintubated video-assisted thoracic surgery. Curr Opin Anaesthesiol.

[24] Hobai IA, Chhangani SV, Alfille PH (2012). Anesthesia for tracheal resection and reconstruction. Anesthesiol Clin.

[25] Zardo P, Kreft T, Hachenberg T (2016). Airway management via laryngeal mask in laryngotracheal resection. Thorac Cardiovasc Surg Rep.

[26] Schieren M, Egyed E, Hartmann B, Aleksanyan A, Stoelben E, Wappler F, Defosse JM (2018). Airway management by laryngeal mask airways for cervical tracheal resection and reconstruction: a single-center retrospective analysis. Anesth Analg.

[27] Kashii T, Nabatame M, Okura N, Fujinaga A, Namoto K, Mori M, Tsujimura S (2016). Successful use of the i-gel and dexmedetomidine for tracheal resection and construction surgery in a patient with severe tracheal stenosis, Masui. Jpn J Anesthesiol.

[28] Cho AR, Kim HK, Lee EA, Lee DH (2015). Airway management in a patient with severe tracheal stenosis: bilateral superficial cervical plexus block with dexmedetomidine sedation. J Anesth.

[29] Umari M, Falini S, Segat M, Zuliani M, Crisman M, Comuzzi L, Pagos F, Lovadina S, Lucangelo U (2018). Anesthesia and fast-track in video-assisted thoracic surgery (VATS): from evidence to practice. J Thorac Dis.

[30] Bertolaccini L, Brunelli A (2019). Devising the guidelines: the techniques of uniportal video-assisted thoracic surgery-postoperative management and enhanced recovery after surgery. J Thorac Dis.

[31] Krecmerova M, Schutzner J, Michalek P, Johnson P, Vymazal T (2018). Laryngeal mask for airway management in open tracheal surgery-a retrospective analysis of 54 cases. J Thorac Dis.

[32] Gao R, Gu X, Zhang S, Ma S, Xu L: Intraoperative airway management for patients with tracheal tumors: A case series of 37 patients. 2021. 10.1111/1759-7714.14181 PMC8590894 34626082

[33] Siciliani A, Rendina EA, Ibrahim M (2018). State of the art in tracheal surgery: a brief literature review. Multidiscip Resp Med.

[34] Yoshida T, Torsani V, Gomes S, De Santis RR, Beraldo MA, Costa EL, Tucci MR, Zin WA, Kavanagh BP, Amato MB (2013). Spontaneous effort causes occult pendelluft during mechanical ventilation. Am J Respir Crit Care Med.

